# Bioprocessing of Epothilone B from *Aspergillus fumigatus* under solid state fermentation: Antiproliferative activity, tubulin polymerization and cell cycle analysis

**DOI:** 10.1186/s12866-024-03184-w

**Published:** 2024-01-30

**Authors:** Ashraf S. A. El-Sayed, Ahmed Shindia, Hala Ammar, Mohamed G. Seadawy, Samar A. Khashana

**Affiliations:** 1 Enzymology and Fungal Biotechnology lab, Botany and Microbiology Department, Faculty of Science, Zagazig University, Zagazig, 44519 Egypt; 2 Biological Prevention Department, Egyptian Ministry of Defense, Cairo, Egypt

**Keywords:** Epothilone, *Aspergillus fumigatus*, Solid state fermentation, Anti-tubulin polymerization, Apoptosis

## Abstract

Epothilone derivatives have been recognized as one of the most powerful anticancer drugs towards solid tumors, for their unique affinity to bind with β-tubulin microtubule arrays, stabilizing their disassembly, causing cell death. *Sornagium cellulosum* is the main source for Epothilone, however, the fermentation bioprocessing of this myxobacteria is the main challenge for commercial production of Epothilone. The metabolic biosynthetic potency of epothilone by *Aspergillus fumigatus,* an endophyte of *Catharanthus roseus,* raises the hope for commercial epothilone production, for their fast growth rate and feasibility of manipulating their secondary metabolites. Thus, nutritional optimization of *A. fumigatus* for maximizing their epothilone productivity under solid state fermentation process is the objective. The highest yield of epothilone was obtained by growing *A. fumigatus* on orange peels under solid state fermentation (2.2 μg/g), bioprocessed by the Plackett-Burman design. The chemical structure of the extracted epothilone was resolved from the HPLC and LC-MS/MS analysis, with molecular mass 507.2 *m/z* and identical molecular fragmentation pattern of epothilone B of *S. cellulosum.* The purified *A. fumigatus* epothilone had a significant activity towards HepG2 (IC_50_ 0.98 μg/ml), Pancl (IC_50_ 1.5 μg/ml), MCF7 (IC_50_ 3.7 μg/ml) and WI38 (IC_50_ 4.6 μg/ml), as well as a strong anti-tubulin polymerization activity (IC_50_ 0.52 μg/ml) compared to Paclitaxel (2.0 μg/ml). The effect of *A. fumigatus* epothilone on the immigration ability of HepG2 cells was assessed, as revealed from the wound closure of the monolayer cells that was estimated by ~ 63.7 and 72.5%, in response to the sample and doxorubicin, respectively, compared to negative control. From the Annexin V-PI flow cytometry results, a significant shift of the normal cells to the apoptosis was observed in response to *A. fumigatus* epothilone by ~ 20 folds compared to control cells, with the highest growth arrest of the HepG2 cells at the G0-G1 stage.

## Introduction

Epothilones are macrolide secondary metabolites that were firstly isolated from the fermentation media of *Sorangium cellulosum* with broad anticancer activity towards various types of tumors [1, 2]. The unique antitumor activity of epothilones and their derivatives elaborates from their higher affinity to interferes with microtubule arrays, binding with β-tubulin, arresting the cells at G2-M phase and stabilizing the tubulin disassembly during the cellular division [2–5]. The mode of action of epothilones is mainly via stabilizing microtubule arrays of tumor cells by, causing cell death [3]. So, both epithilone and Taxol targets the tubulin, halting the functionality of tubulin assembly and disassembly, during mitotic division of cellular growth and replication [5, 6]. However, epothilones have more affordable biological properties and antiproliferative efficiency than Taxol compounds which might be due to their higher water solubility, lower binding energies with tubulin as revealed from the molecular modeling analyses, thus, having a significant effect against the drug-resistant tumors [7]. Due to the higher water solubility of epothilones, it doesn’t require adjuvants for the in vivo applications [4, 5], thus, the antiproliferative activity of epothilones being a thousand fold higher than Taxol towards drug-resistant tumors, especially expressing *P*-glycoprotein [8]. Ixabepilone, a modified derivative of epothilone B was officially approved by the Food and Drug Administration in 2007 [9]. Currently, the commercial epothilones production has been mainly reported from *S. cellulosum*, however, the poor cultivation conditions, and extremely slow growth rate, are the main limiting factors for the industrial production of epothilone, and subsequently their extraordinarily high price, which severely limits their further clinical applications [10]. Under submerged condition, *S. cellulosum* usually form a bacterial clump that hinders the nutrient uptake and metabolite transportation with subsequent suppression of secondary metabolites productivity. So, Epothilone production by *S. cellulosum* is usually occurred by growing on solid medium in presence of resin to scavenger the released metabolites [10]. Biologically, Myxobacteria have a complex social-living pattern by feeding in groups, moving in swarms, germinating myxospores in a cell density manner, and developing multi-cellular fruiting bodies [11]. So, the Epothilone productivity by *S. cellulosum* strongly affected by the co-existing other microorganisms [12].

The biosynthesis of epothilones has been authenticated to be via polyketides synthase type I (PKS) in combination with non-ribosomal peptide synthetase (NRPS) complex (hybrid PK-NRP) [4]. The mixed PKS-NRPS pathway is the most common biosynthetic machinery for the diverse bioactive secondary metabolites due to the formation of amino acids to be incorporated into polyketide backbone [3, 4, 13]. Endophytic fungi with the metabolic ability for epothilone production and feasibility of metabolic manipulating the epothilone biosynthetic machinery raises the hope for commercial production of epothilone [13]. *Aspergillus fumigatus* an endophyte of *Catharanthus roseus,* has the ability to produce epothilone B, with the same chemical structure and antiproliferative activity towards various types of tumor cells of the authentic compound [13]. The productivity of epothilone B by *A. fumigatus* was optimized by the statistical nutritional optimization bioprocess which gave a maximum yield of approximately 55 μg/g fungal biomass [13], that being higher than the yield of *Burkholderia* DSM7029 (8.1 μg/l) by about 6 folds. So, we have been motivated for further experimental studies to maximize the yield of epothilone by *A. fumigatus.*

Solid state fermentation process is one of the well-recognized approaches for maximizing the yield of bioactive secondary metabolites by fungi. Several substrates with organic nature of lignocellulosic biomass which are rich with cellulose, hemicellulose and lignin have been used frequently as solid substrates supporting the fungal growth for production of various bioactive metabolites [14]. The solid-state fermentation process is based on solid matrix within an inert substrate, with very low free water content, that enough to promote the growth and metabolic activity of microorganisms [15]. This fermentation processes usually of reduced production costs due to the lower energy consumption and production area, are proving to be economic and environmentally friendly processes for the production of secondary metabolites from agro-industrial residues [16]. Filamentous fungi and yeasts have been used for the production of various bioactive compounds under solid-state fermentation process [14, 17]. Thus, the objective of this study was to assess the possibility of *Aspergillus fumigatus* for epothilone production using different solid agro-industrial residues as substrates under solid state fermentation bioprocess.

## Materials and methods

### Materials

Different agro-industrial residues such as lemon peels, orange peels, mandarin orange peel, kiwi peels, banana peels and cantaloupe peels were obtained from the local Egyptian markets and used as substrates for growth of *A. fumigatus* for epothilone production. All other chemicals used were of the highest grade available.

### Fungal isolate and culture conditions

The fungal isolate *Aspergillus fumigatus* EFBL, an endophyte of *Catharanthus roseus,* with the highest potency for epothilone production, with Genbank accession # MN744705.1, and deposition # AUMC14078 at Assiut University Mycological Center, Egypt [13]. The fungal culture was maintained on Potato Dextrose Agar [18], incubated at 28 °C for 7 days, stored at 4 °C until use. The epothilone productivity of *A. fumigatus* was assessed by growing on potato dextrose broth (PDB) (Difco, Cat# DF0549-17-9) [13]. The culture was incubated at 30 °C for 15 days, filtered by sterile cheesecloth to collect the fungal biomass. Epothilones were extracted from the fungal mycelia according to [19, 20] with slight modifications. Briefly, five grams of the fresh weight of *A. fumigatus* were homogenized in 30 ml methyl ethyl ketone, incubated overnight at 200 rpm, then filtered to remove the debris of fungal tissues, the solvent was evaporated to dryness and the residue was re-dissolved in 1 ml of dichloromethane. The putative sample of epothilone was fractionated and identified by TLC (pre-coated silica gel plates, silica gel 60 F254, Merck KGaA, Darmstadt, Germany) [13]. The TLC plate was developed with dichloromethane and methanol (95:5) system, and the putative epothilone spots were detected by UV illumination at 254 nm, compared to authentic epothilone B (Cat#. 152,044-54-7), which gave blue colored spots. The intensity of putative spots of epothilone on the TLC plates was assessed by the Image J software package, regarding to the authentic concentrations of epothilone with the same retention time.

### Solid state fermentation media

The agro-industrial residues; lemon peels, orange peels, mandarin orange peel, kiwi peels, banana peels and cantaloupe peels were used as substrates for the growth of *A. fumigatus* for production of epothilone. The chemical composition of these solid byproducts was summarized by [21]. The solid substrates were dried at 50 °C, till constant moisture contents, twenty grams of each substrate were dispensed to 250 ml Erlenmeyer conical flasks, and moistened with 10 ml of salt solution containing 1.0% glucose, 0.25% KH_2_PO_4_, 0.05% KCl and 0.05% MgSO4.7H_2_O of pH 7.0, to get the total moisture contents to approximately 40% [22–25]. The pH of the salt solution was adjusted to 7.0. The flasks were autoclaved, inoculated with 2 ml of *A. fumigatus* spores suspension (10^6^ spore/ ml, of 6 days old), the flasks were mixed thoroughly, incubated for 15 days at 30 ± 1 °C, under static conditions [22]. Control flasks with the same substrates, amended with the same moistening solution of the same salt solution, without fungal spores, incubated at the same conditions were used. After incubation, the solid-state fermented cultures were homogenized in 50 ml methyl ethyl ketone, incubated overnight at 200 rpm, then filtered to remove the debris of solid substrates, then the filtrates were centrifuged at 8000 rpm for 10 min. The supernatant of solvent was evaporated to dryness, and the residue was re-dissolved in 2 mL of dichloromethane. The samples were fractionated by TLC as described above, the putative epothilone spots gave the same color and mobility rate of authentic epothilone B (Cat#. 152,044-54-7) were considered.

### HPLC and LC-MS/MS analyses of the putative epothilone

The putative epothilone-containing silica spots were scraped-off from the TLC plate, dissolved in dichloromethane, vortex for 3 min, centrifuged for 5 min at 8000 rpm to precipitate the silica particles, and the supernatant containing putative epothilone was analyzed by HPLC [13, 26]. The purity and concentration of epothilone was determined by HPLC (YOUNG In, Chromass, 9110+ Quaternary Pump, Korea) with RP-C18 column (Eclipse Plus C18 4.6 mm × 150 mm, Cat. #959963-902), with mobile phase methanol/ water (7030 v/v) at a flow rate 1.0 ml/min for 20 min, scanned by photodiode array detector (DAD). The concentration of the putative epothilone sample was confirmed from retention time and peak area, compared to the authentic one at λ249 nm [27–29].

The FT-IR spectra of the purified sample was analyzed by Bruker FT-IR Spectrometer in range of 400-4000 cm^−1^ with KBr pellets. The sample was dissolved in CDCl_3_. The chemical shifts and coupling constants are expressed in part per million (δ-scale) and hertz (Hz). The purity, molecular mass and identity of the putative epothilones were analyzed by the liquid chromatography-tandem mass spectrometry (LC-MS/MS), and a Thermo Scientific LCQ Deca mass spectrometer equipped with an electrospray source operated in positive-ion mode [30, 31]. The mobile phase A was water containing 0.1% formic acid, and mobile phase B was acetonitrile with 0.1% formic acid. The sample was injected (μl) to a Thermo Scientific Hypersil Gold aQ (C18 column). The compounds were eluted from the column using a gradient of 2–98% mobile phase B over 30 min, with a flow rate of 0.2 mL/min, total run time 40 min. The electrospray ionization (ESI) source was operated with a spray voltage of 4 kV and a capillary temperature 250 °C. For LC-MS, the ion trap was scanned from m/z 300–2000 in positive-ion mode. The scan sequence of the mass was programmed for a full scan recorded between 300 and 2000 Da and an MS/MS scan to generate product ion spectra to determine fragmentation ions in consecutive instrument scans of the four most abundant peaks in the spectrum. The chemical identity of the components was identified based on their mass spectra fragmentation pattern and retention time with NIST mass spectral library.

### Effect of inoculum size, moisture content and particles size of orange peels on *A. fumigatus* epothilone productivity

The effect of the inoculum sizes, moisture content, and particles sizes of the orange peels on the growth and productivity of epothilone by *A. fumigatus* has been estimated [17, 24, 25] under solid state fermentation. Various concentrations of *A. fumigatus* conidial suspensions (10^2^ to 10^5^) per ml were amended to sterilize cubical orange peels (1 cm × 1 cm), moistened with 10 ml salt solution as mentioned above. The cultures were incubated for 15 days at 30 ± 1 °C, under static conditions. Standard media without fungal inocula, incubated at standard conditions, were used as negative controls. After incubation, the cultures were homogenized in methyl ethyl ketone, and epothilone was extracted, and determined by TLC and HPLC as mentioned above.

The effect of moisture content of the orange peels as substrate for growth *A. fumigatus* for production of epothilone under solid state fermentation was determined. The solid substrate “orange peels” were dried at 50 °C until constant weight, amended with different volumes of the salt solution to give the moisture contents with 40-90%, autoclaving, then inoculated with the fungal spores (2 ml spores/20 g medium/250 ml Erlenmeyer conical flask) [14, 16, 17, 24, 25]. After incubation, epothilone was extracted and determined by TLC and HPLC, as mentioned above.

The influence of particle size of the orange peels (0.5 cm, 2.0 cm and 4 cm) on epothilone productivity by *A. fumigatus* was assessed. The media were amended with the salt solution to get the desired moisture content, then autoclaved, and inoculated with the fungal spore suspension. After incubation at the standard conditions, the epothilone was extracted, quantified by TLC and HPLC.

### Bioprocessing of epothilone production by *A. fumigatus* with Plackett-Burman design and faced central composite designs

Various carbon and nitrogen sources (starch, dextrin, lactose, sucrose, soytone, and yeast extract, methyloleate, and sodium butyrate), metal ions (Zn^2+^, Co^2+^, Fe^3+^, Mg^2+^, and Ni^2+^), inhibitors (lamifin, fluconazole, and grisofulvine) at pHs 5 and 10, were optimized by Plackett-Burman design to maximize the yield of epothilone by *A. fumigatus* [13]. The solutions were amended to the orange peels at 50% moisture content, inoculated with *A. fumigatus* spore suspension, and the solid state fermented cultures were incubated for 15 days at static condition. The nineteen variables for the Plackett-Burman design, were represented by high (+ 1) and low (− 1) levels. The design of Placket-Burman depends on the first order reaction: Y = β0 + ΣβiXi where, Y is the predicted epothilone production, Xi is an independent variable, βi is the linear coefficient, and β0 is the model intercept. The runs were conducted in triplicates and the average of epothilone production was used as response. The most significant independent variables affecting epothilone production by *A. fumigatus* was optimized by the faced central composite design (FCCD) to determine the individual and mutual interactions of the tested variables. In the FCCD experimental design, each variable was represented by three different levels low (− 1), medium (0) and high (+ 1) resulting in a total 20 runs [28, 29, 32–34].

### Metabolomics analysis

The fungal biomass (50 mg) was dispensed in 2 ml of working solution (water: methanol: acetonitrile, 2:1:1), vortex for 2 min, then sonication at 20-30 kHz for 10 min. The solution was centrifuged at 10000 rpm for 5 min, then 10 μl at concentration 1 μg/μl, was injected to the Axion AC system (Kyoto, Japan) with X Select HSS T3 (2.5 μm, 2.1 × 150 mm) column maintained at 40 °C, at flow rate 300 μl/min. The mobile phase consists of solution A (5 mM ammonium formate in 1% methanol of pH 3.0), solution B (acetonitrile) and solution C (5 mM ammonium formate in 1% methanol with pH 8.0) [35, 36]. Mass spectrometry was performed on a Triple TOF-TM 5600+ system quadrupole-TOF mass spectrometer. The voltage floating and voltages were + 4500 and + 80 V in positive mode and − 4500 and - 80 V in negative mode. Batches of MS and MS/MS data collection were created by Analyst TF 1.7.1. The metabolites of *A. fumigatus* were annotated and identified using KEGG database by performing the metabolic pathway enrichment analysis of the differential metabolites. The structure information and mass fragments were considered for further validation of the metabolite identities, the online mapper of KEGG library was used to elucidate the differential metabolic pathways (Kanehisa and Goto, 2000).

### Antiproliferative activity of the extracted Epothilone from *A. fumigatus*

The antiproliferative activity of the purified Epothilone from *A. fumigatus* CPT was evaluated towards the liver carcinoma (HepG-2), breast carcinoma (MCF7), pancreatic duct epithelioid carcinoma (PANC-1) and human fetal lung fibroblast (WI-38) by the MTT assay [37]. The cell lines were obtained from American Type Culture Collection, cultured on DMEM (Invitrogen/ Life Technologies), supplemented with 10% FBS (Hyclone), 10 μg/ml of insulin and 1% penicillin-streptomycin. The 96-well microtiter plate were seeded with the cells at density 10^3^ cells/ well in a volume of 100 μl complete growth medium, incubated overnight at 37 °C, then amended with different concentrations of the extracted EPT samples, then further incubated for 48 h at the same conditions. The MTT reagent was added, the plates were incubated for 6 h, and the developed formazan complex with purple color was measured at λ_570_ nm. The IC_50_ value was expressed by the compound concentration reducing the growth of 50% of the initial number of cells, compared to the controls (without drug).

### Wound healing of tumor cells in response to the purified epothilone

The influence of purified epothilone from *A. fumigatus* on the wound healing, and cell migration ability of the HepG2 tumor cells was assessed [38, 39]. Briefly, the HepG2 cells were seeded at 5 × 10^6^ cells of 12-well culture plate, incubated at 37 °C for 24 h, to get a confluent monolayer growth. Once a confluence of monolayer cells was appeared, a wound/ scratch in a straight line with 1 mm pipette tip were made. The monolayer cells were gently washed to remove the detached cells, then replenished with fresh medium containing the extracted epothilone sample with approximately IC_25_ value. Doxorubicin has been used with the same concentrations as the positive control, under the same conditions. The plates were incubated at 37 °C, at for 24 h at the standard incubation conditions. The wound closure due to the cell migration was monitored and imaged by the phase-contrast microscopy. The wound healing percentage was determined based on the area of gap of tumor cells in response to the compound treatment, compared to DMSO treated cells, as control.

### Tubulin polymerization assay

The effect of extracted epothilone from *A. fumigatus* on tubulin polymerization has been assessed with the fluorescence-based tubulin polymerization assay (Cytoskeleton-Cat.# BK011P) [40, 41], according to the manufacturer’s protocol. Tubulin composed of a heterodimer of two closely related 55 kDa proteins (α and β tubulins), isolated from Porcine brain tissue that highly homologous to tubulin of mammalian cells. So, for this technical benefit Porcine tubulin (in the form of microtubules) can be used to assay proteins originating from many diverse species. Polymerization is followed by fluorescence enhancement due to the incorporation of a fluorescent reporter into microtubules as polymerization occurs. The standard assay uses neuronal tubulin (Cat. # T240), generating a polymerization curve representing the three phases of microtubule formation, namely nucleation (Phase I), growth (Phase II) and steady state equilibrium (Phase III). Briefly, 250 μl of the pure tubulin protein was re-suspended in 500 μl of ice cold Tubulin Polymerization Buffer (TPB) (80 mM PIPES (pH 6.9), 2 mM MgCl2, 0.5 mM EDTA, and 1.0 mM GTP) to give a final concentration of 4.0 mg/mL. The tubulin mixture (100 μl) was added to the wells of 96-well plate containing the different concentrations of the extracted *A. fumigatus* epothilone. Paclitaxel was used as positive controls (10 μM). The samples were mixed well and the tubulin polymerization was measured by continuous monitoring of the turbidity change at λ_340_nm (VersaMax™).

### Apoptosis analysis of HepG2 in response to *A. fumigatus* epothilone

The apoptosis analysis of the HepG-2 cell was detected using Annexin V-FITC Apoptosis Detection Kit (Catalog #: K101-25) according to the manufacturer’s instructions. The Annexin V Apoptosis assay based on the observation with the initiation of apoptosis process, the cells translocate the membrane phosphatidylserine (PS) from the inner face of the plasma membrane to the cell surface that can be easily detected by staining with a fluorescent conjugate of Annexin V, a protein with high affinity for PS. The Annexin V-PS interaction can be easily analyzed by flow cytometry. Briefly, the HepG2 cells were seeded in a 96-well plate culture (2 × 10^6^ cells/well), treated with different concentrations of purified epothilone and incubated for 48 h. The cells were harvested and washed with 1 ml phosphate buffered saline (PBS), then adding 200 μl 1X annexin-binding buffer. Annexin V-FITC (10 μl) and PI (10 μl) were added to each 200 μl of cell suspension, and incubated in total darkness for 15 min at room temperature. Annexin-binding buffer (500 μl) was added just before the flow cytometry analysis. The Annexin V-FITC binding was detected by flow cytometry (Ex, 488 nm; Em, 530 nm) with FITC signal detector and PI staining by the phycoerythrin emission signal detector.

### Cell cycle analysis of HepG2 in response to *A. fumigatus* epothilone

The cell cycle analysis of the HepG2 in response to the purified epothilone of *A. fumigatus* was performed by propidium Iodide (PI) Flow Cytometry Kit assay (Cat#. ab139418) according to the manufacturer’s instructions. Briefly, cells were seeded in a 48-well microtiter plate incubated for 12 h at 37 °C, then amended with the IC_25_ values of the extracted epothilone and continue incubated for 48 h. The cells were harvested and centrifuged for 5 min at 2000 rpm, and the cells were then fixed in 1 ml of ice-cold 70% ethanol for 2 h at 4 °C. The fixed cells were rehydrated with 1 ml PBS, and stained with 500 μl of PI containing 5 μg/ml RNase, for 30 min at room temperature in dark. DNA content of the cell was analyzed by flow cytometry at Excitation λ493 nm and Emission λ636 nm. The percentage of G0-G1, S and G2-M cells were then calculated using Fluorescence-activated cell sorting (FACS) software.

### Statistical analysis

The experiments were conducted in biological triplicates, and the results were expressed by the means ± SD. The significance and F-test were calculated using one-way ANOVA with Fisher’s Least Significant Difference of post hoc test.

## Results

### Screening for various agricultural residues supporting the epothilone production by *A. fumigatus*

**Fig. 1:**
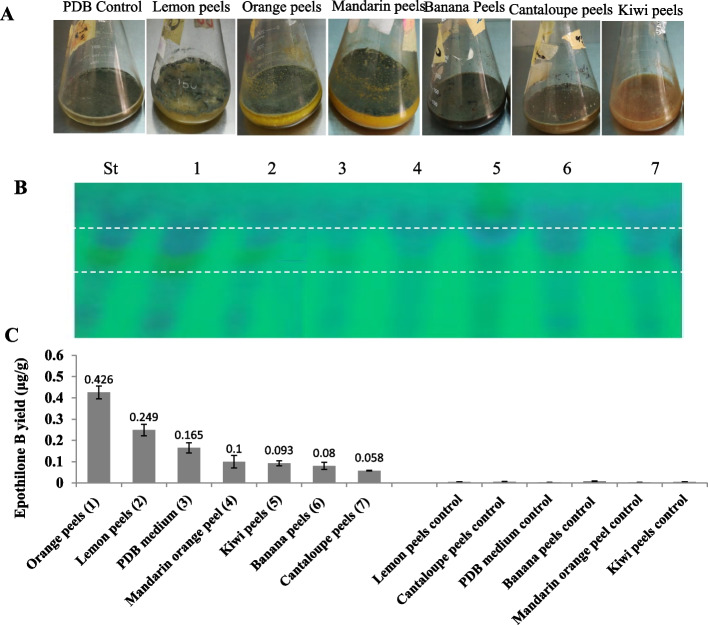
Screening for Epothilone production by *A. fumigatus* grown on different agro-industrial byproducts under submerged conditions. **A**, Submerged cultures of *A. fumigatus* grown on various substrates. **B**, TLC plate for the epothilone extracts from the different media, normalizing to authentic one. **C**, Epothilone yield from the TLC plate as determined by the Image J software, compared to the authentic Epothilone B

**Fig. 2:**
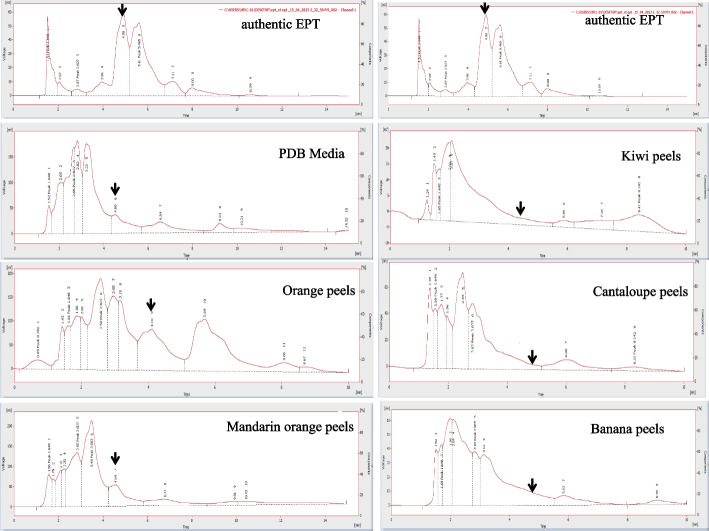
HPLC chromatograms of the extracts of *A. fumigatus* grown on different substrates; orange peels, Mandarin orange peels, Kiwi peels, Cantaloupe peels and Banana peels, compared to the PDB as normal medium. The arrow refers to the predicted peak of the putative epothilone of *A. fumigatus* with the same retention time of authentic epothilone B at 4.5 min

The productivity of epothilone by *A. fumigatus* grown on homogenates of various agricultural residues namely; lemon peels, orange peels, mandarin orange peel, kiwi peels, banana peels and cantaloupe peels, under submerged fermentation conditions, were determined. After incubation, the entire submerged cultures were extracted with methyl ethyl ketone, and the yield of epothilone was checked and quantified by TLC and HPLC as described above. The putative spots of epothilone gave the same mobility rate and the color of authentic epothilone on TLC was quantified by the Image J software (Fig. 1). The yield of epothilone was further quantified by HPLC, as revealed from the retention time and known concentration of authentic epothilone (Fig. 2). From the TLC and HPLC analyses, the maximum yield of epothilone by *A. fumigatus* was obtained with orange peels (0.43 μg/g), followed by lemon peels (0.25 μg/g), mandarin orange peels (0.1 μg/g), compared to potato dextrose broth (0.16 μg/L), as control medium (Fig. 1). The chemical composition of the orange peels and lemon peels with the approximate amounts of the flavonoids, and terpenoids were summarized in Table 1. Negative controls of each agricultural residue without fungal inocula were used under the same conditions. However, the agricultural residues; cantaloupe peels, banana peels, and kiwi peels didn’t support the biosynthesis of epothilone by *A. fumigatus*. Although the obvious visual growth of *A. fumigatus* on all of the tested substrates, however, the physiological behavior and metabolic pattern for epothilone production were obviously different. The supportive effect of orange and lemon peels on the biosynthesis of epothilone by *A. fumigatus*, could be due to the presence of unique flavonoids, and terpenoids as precursors or intermediates of secondary metabolites by *A. fumigatus*. From the TLC and HPLC profiles, orange peels seem the most promising substrate supporting the productivity of epothilone by *A. fumigatus* under submerged fermentation conditions*,* thus*,* further optimization conditions were conducted using orange peels as substrate.

**Table 1:** The chemical composition of these solid byproducts

Lemon Peels			Orange Peels		
Compound	Conc. (%)		Compound	Conc. (%)	
Llimonene	55.40	Paw et al., 2020	β-Linalool	5.6	Qiao et al., 2008
Neral	10.39		δ-Amorphene	0.05	
*Trans*-verbenol	6.43		β-Myrcene	3.3	
Decanal	3.25		Neral	1.3	
Ethyl cinnamate	2.21		δ-3-Carene	0.18	
Ethyl p-methoxycinnamate	2.21		β-Citral	0.15	
*Cis*-α-bergamotene	1.60		Nonanal	0.1	
Geraniol	1.48		L-(+)-Citronellal	0.1	
*Trans*-carveol	1.33		cis-β-Ocimene	0.26	
nonanal	1.19		α-Copaene	0.04	
linalool	1.16		Octanal	0.8	
α-terpineol	1.07		α-Cubebene	0.26	
p-mentha-2,8-dien-1-ol	0.90		(+)-Sabinene	1.0	
Estragole	0.73		γ-Terpinene	1.21	
α-fenchene perillol	0.46		Geranial	1.8	
β-curcumene	0.45		Decanal	0.4	
*trans*-d-limonene oxide	0.43		α-Pinene	0.59	
1-naphthalenamine	0.42		α-Terpineol	0.42	
Camphor	0.34		D-Limonene Ca.	95	
3-carene	0.34		α-Phellandrene	0.07	
β-santalene	0.27				
β-sesquiphellandrene	0.25				
α-pinene	0.17				
terpinen-4-ol	0.17				

### Influence of moisture contents, inoculum size and particles size of orange peels on epothilone productivity by *A. fumigatus*

**Fig. 3:**
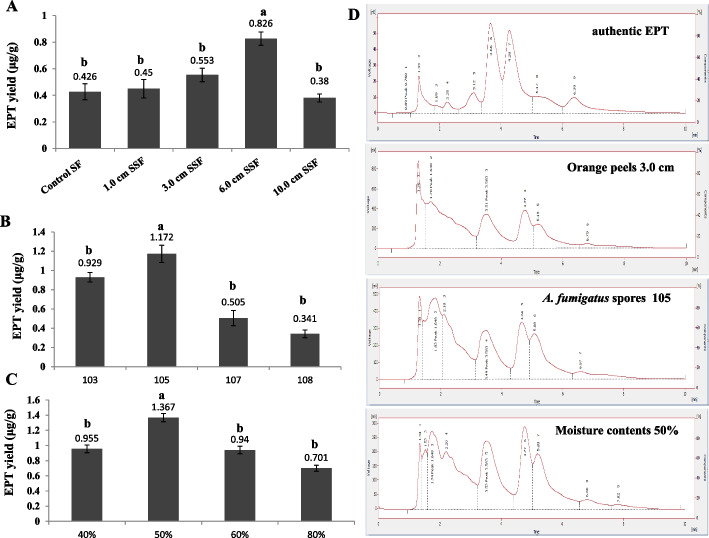
Epothilone yield from *A. fumigatus* grown on orange peels as substrate under solid state fermentation, in response to particle sizes, inoculum sizes and initial moisture contents. **A**, The concentration of the putative Epothilone in response to the particle sizes of orange peels and as homogenate. The concentration of putative epothilone concentration by *A. fumigatus* in response to different inoculum sizes (**B**) and in response to the initial moisture contents (**C**). **D**, HPLC chromatograms of the most potent parameters affecting epothilone production by *A. fumigatus*. The values were represented by the means, followed by letters a, b within the same column that is a significantly different (ONE Way ANOVA, LSD test, *p* ≤ 0.05)

From the screening paradigm, orange peel homogenates were the favored substrate supporting the epothilone production by *A. fumigatus* under submerged fermentation conditions. So, the epothilone productivity by *A. fumigatus* on orange peels was further optimized regarding to their moisture contents, under solid state fermentation conditions. The fungus was grown on orange peels homogenate under submerged condition, as well as on orange peels with particle size 3.0 cm and 50% initial moisture contents under solid state fermentation for 15 days at 30 °C. The initial moisture contents of orange peels were calculated by drying a known weight of orange peels overnight at 60 °C [24]. After incubation, epothilone was extracted and quantified by the TLC and HPLC, as above. From the results (Fig. 3), the yield of epothilone B by *A. fumigatus* under solid state fermentation was increased into 0.83 μg/g, compared to the submerged cultures (0.43 μg/g), i.e. by about 2 folds increments. The influence of particle sizes of orange peels on the growth and epothilone productivity by *A. fumigatus* was investigated. The orange peels with different sizes (1.0 cm, 3.0 cm, 6.0 cm and 10 cm) were amended with the moistening salt solution at 60% initial moisture contents, incubated for 15 days at 30 °C, then epothilone was extracted, quantified by TLC and quantified by HPLC. From the results (Fig. 3), the highest epothilone yield by *A. fumigatus* was obtained at particle size of orange peels 6.0 cm (0.83 μg/g), with about 1.9 folds increments over the control of 1.0 cm orange peels size (0.4 μg/g). The higher productivity of epothilone by *A. fumigatus* at higher particle size might be attributed to the suitability particle size at this moisture contents, giving an optimum micro-environment for maximum growth and highest productivity of epothilone by *A. fumigatus.*

The effect of inoculum size of *A. fumigatus* on its epothilone productivity was assessed. The solid orange peels substrate (6.0 cm) was amended with 2 ml of different concentrations of *A. fumigatus* spores (10,^3^ 10,^5^ 10^7^ and 10^8^ spore/ml), moistened with salt solution at 60% initial moisture contents. The cultures were incubated, under standard conditions, and the epothilone was extracted and quantified by TLC and HPLC. From the results (Fig. 3), a noticeable increasing on the productivity of epothilone by *A. fumigatus* with the increasing on the inoculum sizes. The highest epothilone productivity (Fig. 3B) was obtained at initial spores concentration 10^5^ spore/ml (1.17 μg/g), compared to 10^3^ spore/ml (0.93 μg/g) as control. The rate of visual growth of *A. fumigatus* on orange peels solid substrate was proportionally increased with size of inoculum that partially matched with the productivity epothilone by *A. fumigatus*. The lower metabolic productivity of epothilone at lower spore concentration of *A. fumigatus* might be due to the decreasing of inoculum threshold that would require more incubation time for maximum metabolic activity. The remarkable reduction on the epothilone productivity of *A. fumigatus* with higher inoculum sizes, might be due to the competition for nutrients, or association of self-inhibitory compounds with the spores.

The impact of initial moisture contents on epothilone production by *A. fumigatus* was assessed. The orange peels substrate of 6.0 cm was amended with different volumes of the moistening solution to get the desired initial moisture contents 40%, 50, 60 and 80%. The medium was inoculated with 2 × 10^5^ spores/ml, incubated for 15 days at 30 °C, then epothilone was extracted and quantified by TLC and HPLC. From the results (Fig. 3C), the moisture content has a significant effect on the physiological behavior and metabolic activity of *A. fumigatus*, as revealed from the yield of epothilone. The maximum yield of epothilone by *A. fumigatus* was obtained at initial moisture contents 50% (1.37 μg/g), with significant reduction to the epothilone yield by about two folds at 80% moisture contents (0.71 μg/g), by about 2 folds. The lower yield of epothilone at higher moisture contents might be due to the aggregation of substrate particles, reduction of aeration, causing anaerobic conditions that collectively suppress the fungal growth and productivity of epothilone. The yield of epothilone by *A. fumigatus* for the optimum particle size, inoculum size and moisture content were quantitatively determined by HPLC (Fig. 3D).

### Bioprocess of epothilone production by *A. fumigatus* with the Plackett-Burman design

**Fig. 4:**
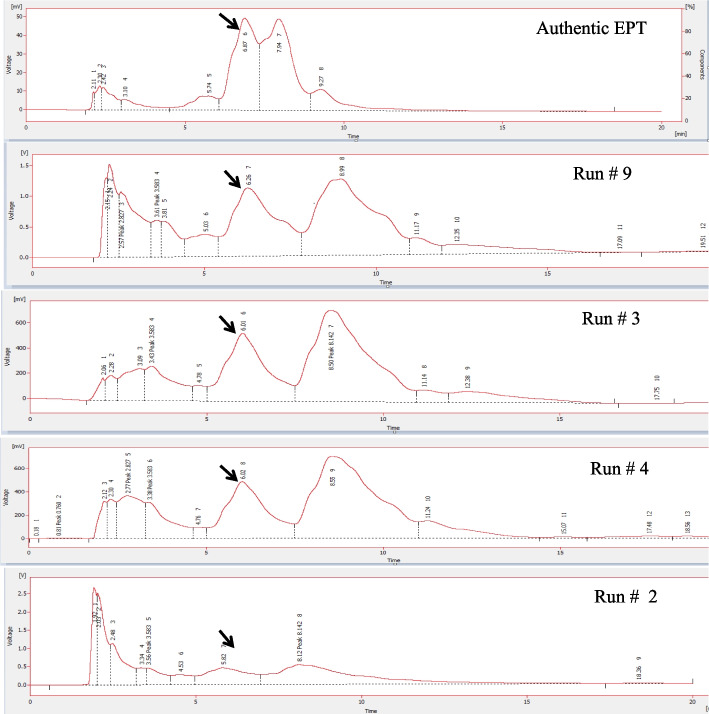
HPLC chromatogram of the selected runs from Plackett-Burman design for optimization of epothilone production by *A. fumigatus* grown on orange peels under solid state fermentation. The highest run (#9) for epothilone production, followed by run # 3, 4 and #2. The arrow refers to the putative peak of epothilone that is correspondent to the retention time of authentic epothilone B, which is at 6.5 min

**Fig. 5:**
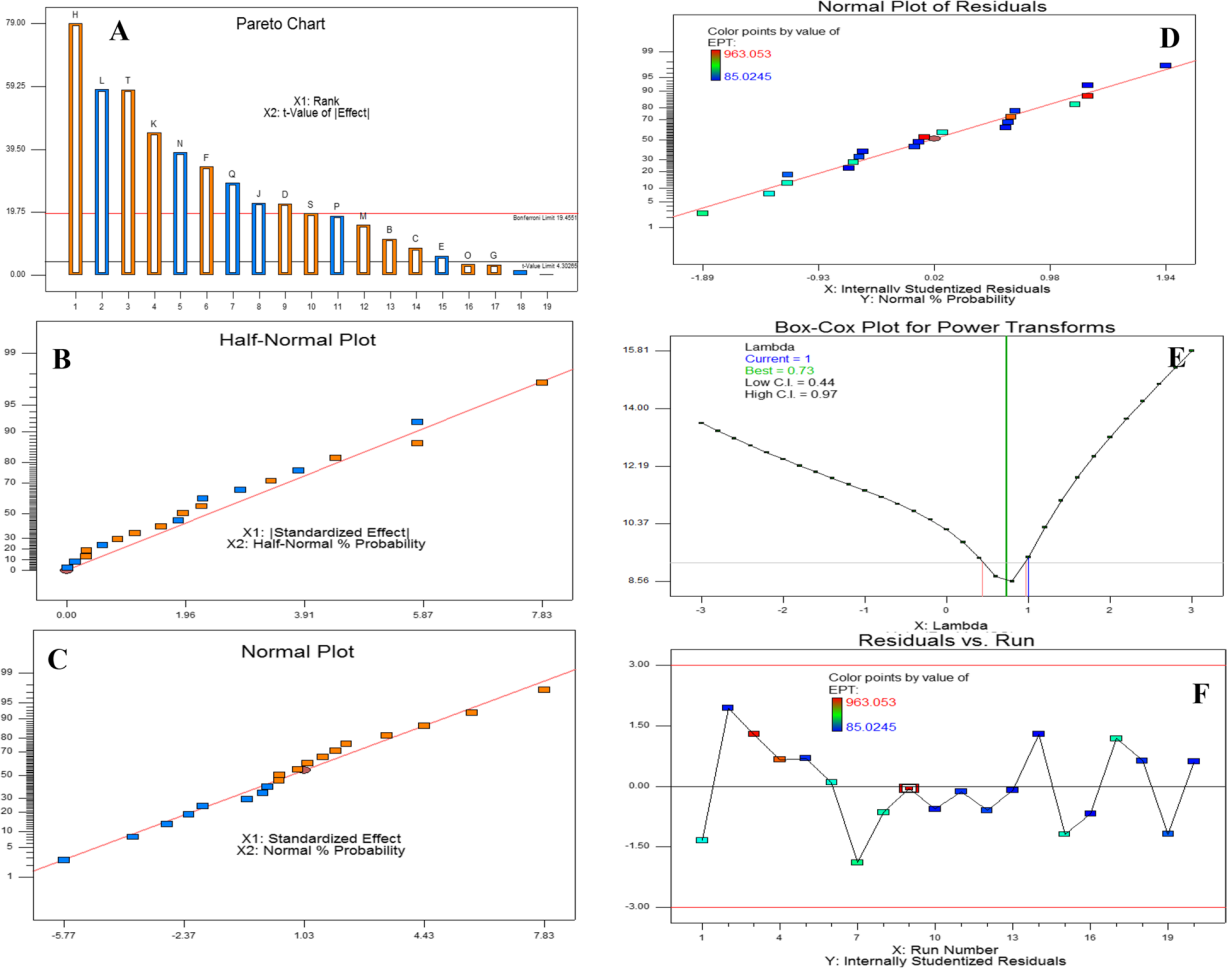
Bioprocess optimization of Epothilone production by *A. fumigatus* under solid state fermentation, with the Plackett-Burman experimental design. **A** Pareto chart illustrating the significance of each variable. Half-normal plot (**B**), normal plot (**C**), normal plots of the residuals (**D**), and Box-Cox power transform (**E**) of epothilone production by *A. fumigatus*. F, Plot of correlation of the predicted and actual camptothecin yield of *A. fumigatus*

Orange peels have been selected as substrate for the production of epothilone by *A. fumigatus* under solid state fermentation process, and some of the physical parameters were optimized as reported above. Further nutritional optimization with response surface methodology by Plackett-Burman design, using the moistening solution at optimum moisture contents, particle sizes, and inoculum sizes, amended with various carbon, nitrogen, growth elicitors/ inhibitors, were conducted to maximize the yield of epothilone by *A. fumigatus.* Since the identity of medium chemical components and their interactions are pivotal for synthesis of secondary metabolites by fungi. The implementing of multifactorial optimization bioprocess is very helpful for assessing not only the identity of chemical components, but also the interaction of these components. The nutritional requirements for epothilone by *A. fumigatus* using orange peels under solid state fermentation process were optimized by Plackett-Burman design as “1st order model equation” to determine the significant factors affecting epothilone production. The tested variables including different carbon, nitrogen sources, in addition to the growth elicitors and inhibitors were studied with their lower (− 1) and higher (+ 1) values (Table 2). After incubation of the solid-state fermented cultures of *A. fumigatus* on orange peels, epothilone was extracted and quantified by TLC and HPLC (Fig. 4). The effect of the independent variables on the productivity of *A. fumigatus* epothilone was summarized in Table 2, as revealed from the predicted and actual responses. The coefficient determination value (R2 = 0.98) indicates the goodness-of-fit of the linear regression models, and the analysis of variance (ANOVA) was calculated, and the t-Stat, *p*-value, and confidence levels were recorded (Table 3). The *F*-value (9.87) and *p*-value (< 0.0007), and adjusted determination coefficient (R2 = 0.92) reveals the significance of this model. The main effects and the normal probability of the tested factors were plotted, and the eight independent factors; sucrose, yeast extract, salicylic acid, Zn^2+^,Co^2+^, Fe^2+^, Mg^2+^ and lamifine, were the most significant factors affecting epothilone productivity. The other independent factors have no obvious effect on epothilone production by *A. fumigatus*. From the Plackett-Burman design matrix (Table 4), the maximum actual and predicted yields of epothilone by *A. fumigatus* were 2.41 μg/g and 2.4 μg/g, respectively, with − 0.01 residuals, using orange peels under solid state fermentation at run # 9. At run # 3, the actual and predicted yield of epothilone by was 2.38 and 2.31 μg/g, respectively, with the residuals 0.07. However, the lowest concentration of actual yield (0.22 μg/g) and predicted yield (0.14 μg/g) of epothilone by *A. fumigatus* was reported at run# 14. The noticeable fluctuation on the epothilone yield by *A. fumigatus* with orange peels as substrate under solid state fermentations reveals the significance of the Plackett-Burman design. The efficiency of the Plackett-Burman design for bioprocessing the epothilone production by *A. fumigatus* was revealed from the Pareto Chart, half-normal plot, normal plot, normal plot of residuals, Box-Cox power transform (Fig. 5). The arrangement of the residuals points around the diagonal line shows the independent normal distribution of variables, suggesting the perfect fitting of the design on the epothilone yield. From the ANOVA analysis, the model was highly significant as shown from the Fisher’s F-test 9.87 and probability *p*-values 0.0007. Thus, the actual and predicted yield of epothilone *A. fumigatus* was noticeably fluctuated from 2.4 to 0.21 μg/g, this confirm the significance of the tested variables on epothilone biosynthesis and the efficiency of the Plackett-Burman design. The values of coefficient of determination (R2 = 0.98) indicating the goodness-of-fit measure for the linear regression models (Table 4). The first order polynomial equation of epothilone production by *A. fumigatus*, considering the significant independent variables, was as follows;

$$\textrm{Epothilone}\ \textrm{yield}\ \left(\upmu \textrm{g}/\textrm{g}\right)=172.5+25.2\ast \textrm{Lactose}+43.3\ast \textrm{Sucrose}+31.1\ast \textrm{Yeast}\ \textrm{Extract}+380.1\ast \textrm{Zn}+-103.6\ast \textrm{Co}++245.9\ast \textrm{Fe}2+-302.2\ast \textrm{Mg}+84.3\ast \textrm{Ni}-184.5\ast \textrm{Lamifine}-143.3\ast \textrm{Methyloleate}-50.2\ast \textrm{Sodium}\ \textrm{butyrate}$$

**Table 2:** Coded values of the Plackett-Burman Design for epothilone production by *Aspergillus fumigatus* under solid state fermentation

code	variable	Level	
		-1	+1
X1	Starch (g/L)	2	5
X2	Dextrin (g/L)	2	4
X3	Lactose (g/L)	1	3
X4	Sucrose (g/L)	2	4
X5	Soytone (g/L)	2	4
X6	Yeast extract (g/L)	2	6
X7	Glucose (g/L)	4	8
X8	pH	5	8
X9	Fluconazole (g/L)	1	3
X10	Zn^+^ (g/L)	0.1	0.5
X11	Co^+^ (g/L)	0.1	0.5
X12	Fe^2+^ (g/L)	0.1	0.5
X13	Mg^2+^ (g/L)	0.2	1
X14	Ni^+^ (g/L)	0.1	0.5
X15	Lamifin (g/L)	1	2
X16	Glisofulvin (g/L)	1	2
X17	Methyloleate (g/L)	0.5	1
X18	Incubation day	10	15
X19	Sodium butyrate (g/L)	1	3

**Table 3:** Matrix of Plackett-Burman Design for epothilone production by *A. fumigatus* under solid state fermentation

Run	X1	X2	X3	X4	x5	X6	X7	X8	X9	X10	x11	X12	X14	X15	x16	X17	X18	X19	Actual	Predicted	Residuals
1	2	4	1	4	2	2	5	0.2	1	1	0.2	1	1	0.5	3	3	10	2	**0.8**	**0.89**	**-0.09**
2	5	4	3	2	4	2	10	0.2	0.2	0.2	0.2	1	1	1	3	1	15	2	**0.26**	**0.152**	**0.108**
3	5	2	3	4	2	2	10	1	1	1	0.2	1	2	0.5	1	1	10	2	**2.38**	**2.31**	**0.07**
4	5	4	1	2	4	6	10	1	0.2	1	0.2	1	1	0.5	1	3	15	0	**2.211**	**2.2**	**0.011**
5	5	4	1	4	4	2	5	1	1	1	1	0.2	1	1	1	1	10	0	**0.8**	**0.83**	**-0.03**
6	5	4	1	4	2	6	5	0.2	0.2	0.2	1	1	2	1	1	1	15	2	**0.85**	**0.85**	**0**
7	2	2	1	2	4	6	5	1	1	0.2	0.2	1	2	1	1	3	10	2	**1.02**	**1.15**	**-0.13**
8	2	4	1	2	2	2	10	1	0.2	1	1	0.2	2	1	3	3	10	2	**0.92**	**0.97**	**-0.05**
9	2	2	3	4	4	6	5	1	0.2	1	0.2	0.2	1	1	3	1	15	2	**2.41**	**2.42**	**-0.01**
10	2	4	3	2	4	6	5	0.2	1	1	1	1	2	0.5	3	1	10	0	**0.25**	**0.29**	**-0.04**
11	2	2	3	4	2	6	10	0.2	0.2	1	1	1	1	1	1	3	10	0	**0.32**	**0.33**	**-0.01**
12	2	4	3	4	4	2	10	0.2	1	0.2	0.2	0.2	2	1	1	3	15	0	**0.37**	**0.37**	**0**
13	2	2	1	2	2	2	5	0.2	0.2	0.2	0.2	0.2	1	0.5	1	1	10	0	**0.25**	**0.25**	**0**
14	5	2	1	2	2	6	10	0.2	1	1	0.2	0.2	2	1	3	1	15	0	**0.22**	**0.15**	**0.07**
15	5	4	3	4	2	6	5	1	0.2	0.2	0.2	0.2	2	0.5	3	3	10	0	**0.89**	**0.96**	**-0.07**
16	5	2	3	2	2	2	5	1	1	0.2	1	1	1	1	3	3	15	0	**0.22**	**0.26**	**-0.04**
17	2	4	3	2	2	6	10	1	1	0.2	1	0.2	1	0.5	1	1	15	2	**0.93**	**0.86**	**0.07**
16	2	2	1	4	4	2	10	1	0.2	0.2	1	1	2	0.5	3	1	15	0	**0.9**	**0.86**	**0.04**
19	5	2	3	2	4	2	5	0.2	0.2	1	1	0.2	2	0.5	1	3	15	2	**0.42**	**0.49**	**-0.07**
20	5	2	1	4	4	6	10	0.2	1	0.2	1	0.2	1	0.5	3	3	10	2	**0.25**	**0.22**	**0.03**

**Table 4:** ANOVA for selected factorial model, Analysis of variance table

	Sum of		Mean	F	p-value	
Source	Squares	df	Square	Value	Prob > F	
Model	1.60E+06	13	1.23E+05	66.4	< 0.0001	significant
C-Lactose	12706.51	1	12706.51	6.84	0.0398	
D-Sucrose	37657.12	1	37657.12	20.28	0.0041	
F-Yeast Extract	77855.6	1	77855.6	41.92	0.0006	
H-Zn^+^	4.62E+05	1	4.62E+05	248.92	< 0.0001	
J-Co^+^	34355.26	1	34355.26	18.5	0.0051	
K-Fe^2+^	1.94E+05	1	1.94E+05	104.22	< 0.0001	
L-Mg^2+^	2.92E+05	1	2.92E+05	157.44	< 0.0001	
M-Ni+	22769.31	1	22769.31	12.26	0.0128	
N-Lamifin	1.70E+05	1	1.70E+05	91.74	< 0.0001	
P-Methyloleate	25697.36	1	25697.36	13.84	0.0099	
Q-Sodium butyrate	50245.56	1	50245.56	27.06	0.002	
S-Incubation time	27695.18	1	27695.18	14.91	0.0083	
T-Shaking	1.96E+05	1	1.96E+05	105.28	< 0.0001	
Residual	11142.63	6	1857.1			
Cor Total	1.61E+06	19

**Fig. 6:**
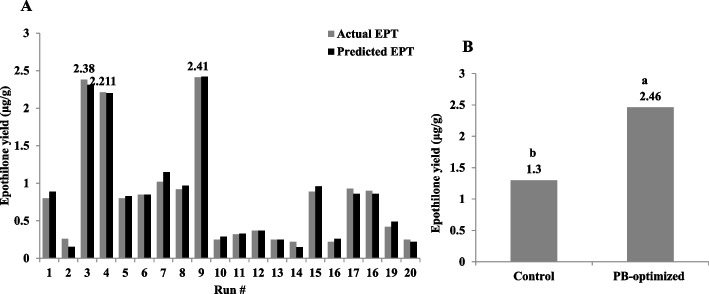
The yield of epothilone by *A. fumigatus* under solid state fermentation, optimized by Plackett-Burman Design. The fungal cultures grown on the corresponding medium components, incubated, and the epothilone was extracted, and quantified. **A**, The yield of epothilone from the TLC sheet as quantified by Image J software. **B**, The overall yield of epothilone by *A. fumigatus* grown on optimized medium components by Plackett-Burman design compared to non-optimized media. The values were represented by the means, followed by letters a, b within the same column that is a significantly different (ONE Way ANOVA, LSD test, *p* ≤ 0.01)

The actual and predicted yield of epothilone *A. fumigatus* which noticeably fluctuated from 2.42 to 2.41 μg/g, ensures the significance of the tested variables and the efficiency of the Plackett-Burman design. The maximum yield of epothilone (run # 9) was obtained using orange peels substrate under solid state fermentation for *A. fumigatus*, moistened with salt solution of lactose (1 g/L), sucrose (2 g/L), yeast extract (2 g/L), Zn^2+^ (0.2 g/L), Fe^2+^ (0.2 g/L), Ni^2+^ (0.2 g/L), lamifin (2 g/L), incubated for 15 days at 30 °C. So, upon using orange peels as substrate, the actual yield of epithilone by *A. fumigatus* under solid state fermentation, upon optimization by Plackett-Burman design was increased into 2.46 μg/g, compared to the basal medium (1.3 μg/g), i.e. by about 1.8 increment folds (Fig. 6).

### Metabolomics profiling of *A. fumigatus* grown on orange peels at solid state fermentation

**Fig. 7:**
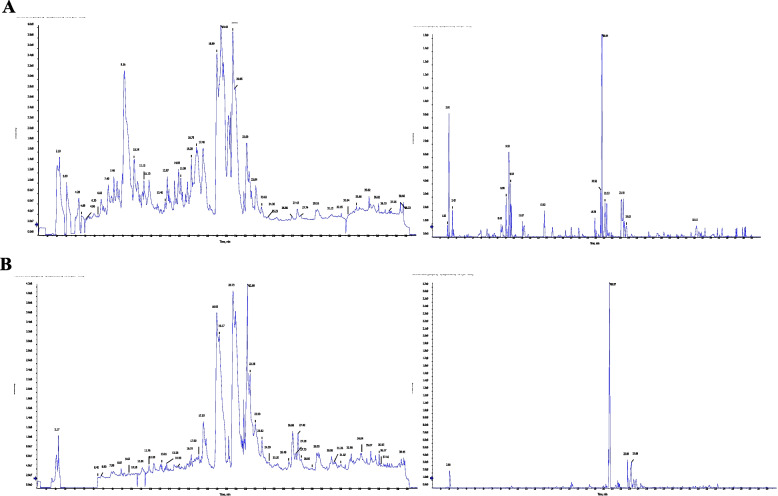
Metabolomic analysis of the of *A. fumigatus* grown on range peels compared to orange peels without fungus as control. **A**, The Total Ion Chromatogram of *A. fumigatus* grown on orange peels as substrate. **B**, The Total Ion Chromatogram of the orange peel without spores of *A. fumigatus* as control

**Fig. 8:**
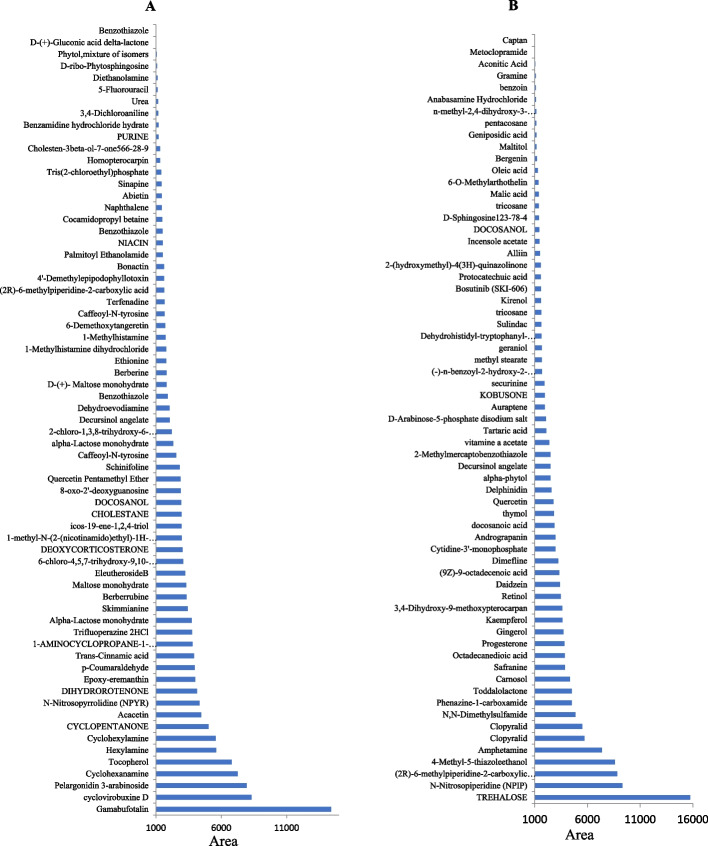
Metabolomic analysis of *A. fumigatus* grown on range peels compared to orange peels without fungus as control. **A**, Metabolic constituents of orange peel without fungal spores, as control. **B**, Metabolic constituents of *A. fumigatus* grown on orange peels as solid substrate

The total ion chromatograms (TIC) of LC-MS/MS acquisitions of *A. fumigatus* grown in orange peels, compared to orange peels without fungus, as control was shown. From the TIC chart, panels of diverse metabolites for the *A. fumigatus* cultures grown in orange peels compared to un-inoculated orange peels as control were shown along the 40 min of separation time (Fig. 7). The metabolites were filtered based on their intensities, relative abundance with cutoff 1000 unit. Among the annotated metabolites, approximately 68 metabolites were differentially fluctuated in the cultures of *A. fumigatus* grown in orange peels compared to control orange peels as substrate, under solid state fermentation, as revealed from the ESI+ and ESI- modes. The differentially abundant metabolites were mainly belongs to flavonoids, alkaloids, disaccharides, sugar amino acids, aromatic amino acids derivatives. The most abundant primary and secondary metabolites of the orange peels without fungus were gamabufotalin, cyclovirobuxine, pelargonidin, 3-arabinosides, cyclohexanamine, tocopherol, hexylamine, cyclopentanone, acacetin, and nitroso-pyrrolidine (Fig. 8A). From the metabolic analysis of the solid fermented cultures of *A. fumigatus* in orange peels, the most abundant metabolites were the trehalose, nitrosopiperidine, 6-methylpiperidine, 4-methyl-5-thiazole-ethanol, amphetamine, clopyralid, dimethylsulfamide, phenazine-1-carboxamide, toddalolactone, carnosol, safranine, octadecanedioic acid, gingerol, progesterone and kaempferol (Fig. 8B). Trehalose was the most abundant disaccharide in the cultures of *A. fumigatus* grown on orange peels under solid state fermentation by about 16 folds increase then the control. From the KEGG database, the differential metabolites were annotated by enrichment analysis to explore their metabolic interactions with the biosynthetic pathway of secondary metabolites, especially epothilone-related biosynthetic pathways, in *A. fumigatus*. The differentially abundant metabolites were mainly involved in the glycolysis, TCA cycle, mevalonate pathway, terpenoids biosynthesis and shikimate pathways, as revealed from the metabolic interactions derived from the KEGG pathway mapper. Practically, the detected metabolites by *A. fumigatus* grown in orange peels under solid state fermentation have a multiple interactions with the glycolysis, TCA cycles, terpenoids, and alkaloids biosynthesis.

### Chemical structure validation of the extracted epothilones by LC-MS/MS analysis

**Fig. 9:**
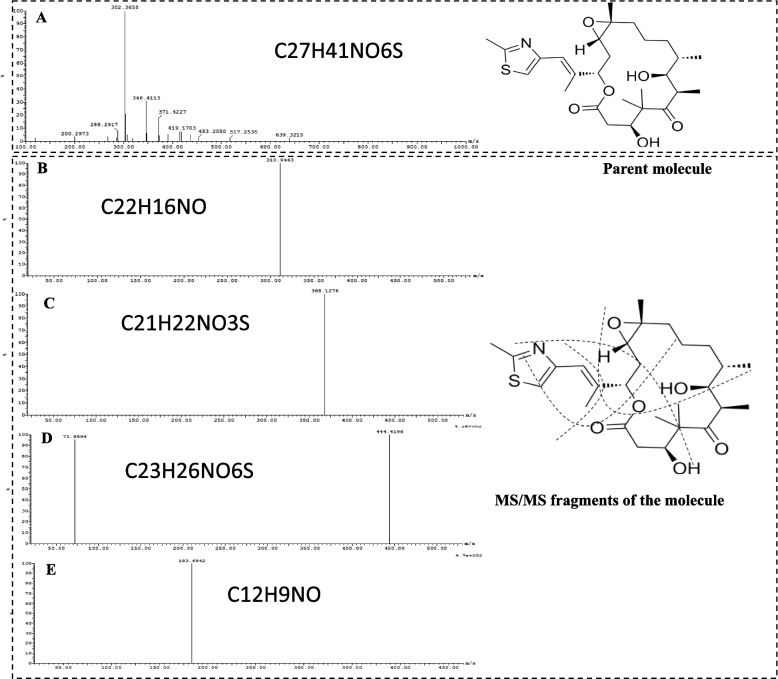
LC-MS/MS analysis of the putative epothilone from *A. fumigatus* grown on orange peels. The putative epothilone spots were scarped off from the TLC and analyzed by LC-MS/MS . **A**, The molecular mass of the parent methylated epothilone molecule was 517.2 *m/z.* .The fragmentation pattern of the parent epothilone molecule into fragments of 310.9 m/z (C22H16NO) (**B**), 368.1 m/z (C21H22NO3S) (**C**), 444.4 m/z (C23H26NO6S) (**D**) and 183.6 m/z (C12H9NO) (**E**)

The putative epothilone samples were basically defined and determined based on the TLC and HPLC analyses, for the above experimental analysis. Further mass spectroscopic analysis by the LC-MS and LC-MS/MS for the putative epothilone samples scraped-off from the silica gel spots, were conducted for more chemical structure validation analysis. The extracted sample from *A. fumigatus* gave molecular mass of 517.2 m/z that mainly attributed to the methyl-epothilone, that being consistent with the molecular mass of un-methylated epothilone B which is 507.4 m/z (Fig. 9). The parent putative *A. fumigatus* epothilone of 517.2 m/z was further fragmented into smaller molecules by the MS/MS analysis. From the fragmentation pattern of the parent epothilone molecule, different fragments of 310.9 m/z (C22H16NO), 368.1 m/z (C21H22NO3S), 444.4 m/z (C23H26NO6S) and 183.6 m/z (C12H9NO), that being consistent with the molecular mass fragmentation patterns of the authentic epothilone B from *Sorangium cellulosum*. Thus, from the TLC, HPLC and molecular mass fragmentation pattern by LC-MS/MS, the putative epothilone sample from *A. fumigatus* grown on orange peels under solid state fermentation had the same chemical structure and molecular mass fragmentation pattern of authentic epothilone B.

### Antiproliferative activity and anti-tubulin polymerization of *A. fumigatus* Epothilone

**Fig. 10:**
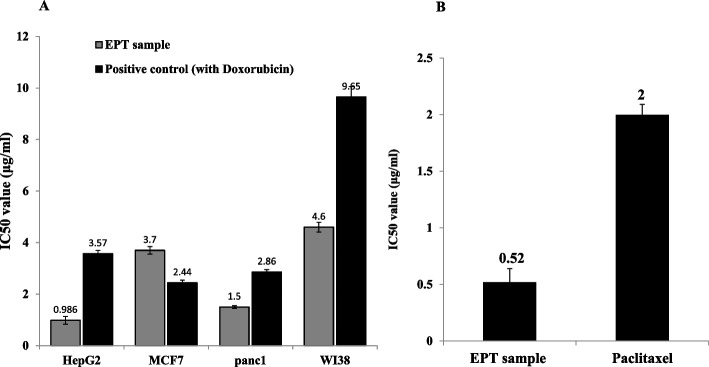
Antiproliferative activity and anti-tubulin polymerization assay of the purified Epothilone from *A. fumigatus.* **A**, The activity of the purified Epothilone was assessed towards various cell lines namely HePG2-, MCF7, Pancl, WI38, and as revealed from the IC50 values. **B**, The anti-tubulin polymerization assay of purified Epothilone from *A. fumigatus*

The activity of purified Epothilone from *A. fumigatus* has been assessed towards the liver carcinoma (HepG-2), breast carcinoma (MCF7), pancreatic duct epithelioid carcinoma (PANC-1) and human fetal lung fibroblast (WI-38) by MTT assay. The viability of the tested cell lines were assessed in response to the different concentrations of extracted epothilone from *A. fumigatus,* compared to doxorubicin as positive control. As revealed from the IC_50_ values (Fig. 10A), the extracted epothilone had a significant activity against the tested cell lines, compared to doxorubicin as an authentic anticancer drug. From the IC_50_ values, the purified epothilone had a significant activity towards HepG2 (0.98 μg/ml), Pancl (1.5 μg/ml), MCF7 (3.7 μg/ml) and WI38 (4.6 μg/ml). From the results, the activity of purified epothilone being much higher than the activity of doxorubicin for the tested cell lines. For the HepG2, Pancl and WI38, the activity of epothilone of *A. fumigatus* was judged to be higher than doxorubicin by about 2 folds, that could be ascribed to their different mode of actions for targeting the tumor cells.

The effect of extracted *A. fumigatus* epothilone on the tubulin polymerization was assessed by the fluorescence-based tubulin polymerization assay. Different concentrations of the extracted epothilone were amended to the tubulin reaction mixture, compared to Paclitaxel as positive control, and the tubulin polymerization was assessed. From the IC_50_ values (Fig. 10B), the purified epothilone displayed a significant activity anti-tubulin polymerization (IC_50_ value 0.52 μg/ml) compared to Paclitaxel (2.0 μg/ml), as standard drug.

### Wound healing of tumor cells in response to purified epothilone of *A. fumigatus*

**Fig. 11:**
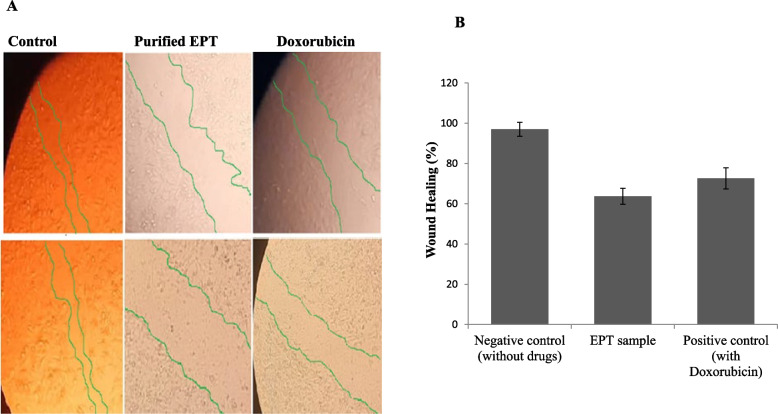
Wound healing assay of the HepG2 cells in response to purified Epothilone from *A. fumigatus* comparing to the untreated cell lines (negative control) and treated with doxorubicin as positive control. After 24 h of growth of MCF-7 as homogenous monolayer, a scratch was made, and the extracted Epothilone was added (IC50 values 3.5 μg/ml), as well as, doxorubicin was added (IC50 values 2.4 μg/ml) to the medium, then the wound healing was measured at zero time after 24 h of incubation (**A**). The percentage of wound healing of the MCF7 cells in response to Epothilone from *A. fumigatus* was calculated (**B**)

The effect of purified *A. fumigatus* epothilone on the cells migration ability of the HepG2 tumor cells was assessed by measuring the wound closure after 24 h, compared to the negative control (untreated) cells, and positive control cells (Doxorubicin treated). From the results (Fig. 11), the gap/wound closure of the HepG2 monolayer cells was significantly suppressed upon using the purified epothilone from *A. fumigatus,* with an obvious similarity to the doxorubicin as authentic anticancer drug. Remarkably, the wound healing of the homogenous monolayer of HepG2 was measured to be about 63.7 and 72.5% in response to purified epothilone sample and doxorubicin, respectively, compared to negative control cells. The suppression of wound healing of HepG2 upon addition of *A. fumigatus* epothilone, confirm the interference with the cellular regeneration, cell divisions, and matrix formation of the tumor cells.

### Apoptosis and cell cycle analysis of HepG2 in response to *A. fumigatus* epothilone

**Fig. 12:**
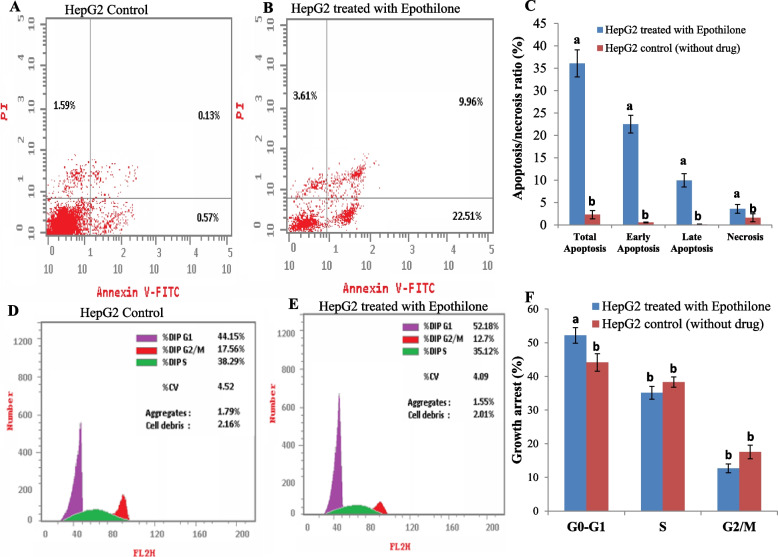
Flow cytometric analysis of apoptosis in HepG-2cells measured by Annexin V-FITC apoptosis detection Kit. The cells were exposed to IC25 concentration of putative epothilone, the apoptosis was measured after 48 h of incubation. Apoptotic analysis of HepG2-cells without epothilone treatment (**A**), and in response to treatment with *A. fumigatus* epothilone (**B**) and overall quantitative results of apoptosis (**C**). The cell cycle analysis of the HepG-2 cells without epothilone treatment (**D**), and in response to treatment with *A. fumigatus* epothilone (**E**) and overall quantitative analysis of cell cycle (**F**). The values were represented by the means, followed by letters a, b within the same column that is a significantly different (ONE Way ANOVA, LSD test, *p* ≤ 0.05)

The effect of the extracted epothilone of *A. fumigatus* on the apoptotic process of the HepG2 cells was assessed by Annexin V -propidium iodide assay. With the initiation of the apoptosis process the membrane phosphatidylserine (PS) that naturally located on inner leaflet of the plasma membrane, was reoriented to be on the outer leaflet of the plasma membrane that easily reacted with Annexin V-FITC, that was easily detected by flow cytometry, elucidating the stages of apoptosis, and necrosis. From the flow cytometry data results (Fig. 12), a significant shift of the normal cells to an early apoptotic and late apoptotic stages, were observed in response to treatment with epothilone of *A. fumigatus,* compared to control cells (untreated cells)*.* From the flow cytometry results, the percentage of the HepG2 cells in early apoptosis, late apoptosis, and necrosis were about 22.5, 9.98 and 3.61%, in response to the epothilone of *A. fumigatus*. However, the negative control cells (without treatment), the percentage of early apoptosis, late apoptosis, and necrosis were 0.57, 0.13 and 1.59%, respectively. Thus, the percentage of total apoptosis of the HepG2 cells was increased by about 20 times in response to treatment with epothilone, compared to the untreated cells.

The cell cycle of the HepG2 in response to the *A. fumigatus* epothilone was analyzed by propidium iodide. The cells were amended with the extracted epothilone of *A. fumigatus* at their IC_25_ values, after incubation the cells were harvested by centrifugation, and then fixed by 1 ml ice cold 70% ethanol for 2 h at 4 °C. The DNA content of the cells was analyzed by flow cytometry, and the percentage of G0-G1, S and G2-M cells were then calculated by FlowJo software. From the cell cycle analysis (Fig. 12), the highest growth arrest of the HepG2 cells was reported at the G0-G1, in response to the treatment with epothilone from *A. fumigatus,* compared to the control cells without treatment. The growth arrest of the HepG2 tumor cells at the S- phase and G2/M phases in response to the treatment with epothilone and negative control cells (without drug treatment) was not significantly different. Overall, the epothilone extracted from *A. fumigatus* displayed significant activity at the G0-G1 cell cycle, as revealed from the maximum growth arrest, compared to the other phases of the cell cycle.

## Discussion

Epothilones derivatives have been recognized with their powerful anticancer activity towards various types of solid tumors (Bollag et al., 1998). The unique activity of epothilones elaborates from their higher affinity to bind with β-tubulin microtubule arrays, stabilizing the tubulin disassembly, causing cell death [3, 9, 42]. The main kinetic advantage of epothilones than the paclitaxel is the higher water solubility, lower binding energies with tubulin as revealed from the molecular modeling analyses, with noticeable effect against the multiple drug-resistant tumors [6, 7]. Currently, *S. cellulosum* has been used as the common source for production of epothilones, however, the extremely slow growth rate and poorly cultivation conditions are the main limiting factors for the industrial production of epothilone based on this bacterial isolate. For the first time, *Aspergillus fumigatus* an endophyte of *Catharanthus roseus,* had been reported to have an efficient metabolic potency for epothilone B production, with the same chemical structure and antiproliferative activity towards various types of tumor cells of the authentic one [13]. The productivity of epothilone B by *A. fumigatus* was higher than the yield of *Burkholderia* sp. by ~ 6 folds. So, we have been motivated for further experimental studies to maximize the yield of epothilone by *A. fumigatus.* Solid state fermentation using the lignocellulosic substrates rich with cellulose, hemicellulose, and lignin, with low free water content, is one of the most common approaches for maximizing the yield of secondary metabolites by fungi. Thus, the objective of this work was to assess the productivity of epothilone by *Aspergillus fumigatus* under solid state fermentation, as well as to evaluate the antiproliferative activity, anti-tubulin polymerization, and cell cycle analysis in response to the purified epothilone.

The productivity of epothilone by *A. fumigatus* grown on homogenates of various agricultural residues was determined under submerged fermentation conditions. The maximum yield of epothilone by *A. fumigatus* was obtained with orange peels (0.426.8 μg/g), followed by lemon peels, compared to potato dextrose broth, as control medium. The favorability of orange peels juice as good support for fungal growth and epothilone productivity might be attributed to their higher constituents of flavonoids, with antioxidant activity that mainly due to hesperidin and naringin [43]. As well as, orange peels are rich with the coniferin, phlorin, and polymethoxylated flavones with free radical scavenging activity [44], could be an excellent elicitors for induction of the epothilone biosynthesis by *A. fumigatus*. In addition, the orange peels have higher amounts of naringin and flavanone glycoside [45] that could induce the antioxidant enzymatic system of *A. fumigatus*, with positive correlation on epothilone biosynthesis. Since, orange peels juice gave the maximum epothilone productivity by *A. fumigatus*, so, we are motivated to use the orange peels as fungal substrate under solid state fermentation that could be have a positive effect on epothilone biosynthesis by *A. fumigatus*. The yield of epothilone by *A. fumigatus* grown on orange peels of particle size 6.0 cm, under solid state fermentation was increased by about 2 folds, compared to orange peels juice under submerged fermentation. From the results, the highest epothilone yield by *A. fumigatus* was obtained at particle size of orange peels 6.0, with about 1.9 folds increments over the orange peels of 1.0 cm size, that might be due to positive interaction of particle size and their moisture contents, gave an optimum microenvironment for productivity of epothilone by *A. fumigatus.*

A noticeable increasing on the productivity of epothilone by *A. fumigatus* with the inoculum size was observed. The moisture content has a significant effect on the physiological behavior and epothilone productivity by *A. fumigatus.* The maximum epothilone yield by *A. fumigatus* was obtained at moisture contents 50%, that being an optimum for particles aggregation, aeration, cell turgor pressure, with the constant branching orientation and hyphal growth rate and subsequent metabolic productivity of epothilone [46, 47]. Interactions among water status, oxygen supply and heat removing from SSF could be the main factors controlling the epothilone productivity by *A. fumigatus.* However, the significant reduction to the epothilone yield at 80% moisture content could be due to the aggregation of particle of substrate, changing the microenvironment to anaerobic that collectively suppresses the fungal growth [48]. Water content is a very significant factor in the fermentation process. High water activity causes the decrease in porosity of the substrate, thereby reducing the exchange of gases, while the low water activity result in reduction of microbial growth and consequent suppression on enzyme productivity [49]. The nutritional requirements for epothilone production by *A. fumigatus* using orange peels under solid state fermentation were optimized by Plackett-Burman design. Response surface methodology (RSM) is an experimental method that is a useful tool to optimize the parameters of fermentation processes by applying mathematics and statistics [11, 24, 25, 30, 47–51]. From the matric of Plackett-Burman design, the actual yield of epothilone *by A. fumigatus* was increased up to 0.96 μg/g by 1.2 folds compared to the control medium. The cultural bioprocessing by the response surface methodology factorial design has been recently used as a common method for optimization of the microbial secondary metabolites productivity, by assessing the variables interactions [51–53].

The activity of the purified *A. fumigatus* Epothilone was assessed against the HepG-2, MCF7, PANC-1, and WI-38 cell lines. The extracted epothilone had a significant activity towards the tested cell lines, compared to paclitaxel. The activity of *A. fumigatus* epothilone for the HepG2, Pancl and WI38, being higher than paclitaxel by ~ 2 folds. The higher activity of the extracted epothilone for the tumor cells than Paclitaxel ensures their broad spectrum activity for the taxane- resistant tumors [3]. Similarly, epothilones have been demonstrated high antitumor activity in vitro against tumor cell lines that are naturally resistant to paclitaxel or acquire resistance to paclitaxel by specific point mutations in the β-tubulin gene [3, 54]. The epothilones are potent in cell culture models than is paclitaxel, with the IC_50_ values in nanomolar range in breast, lung, colon, prostate, and ovarian cell lines as reviewed by [55]. The resistance of SKOV-3 cells to Paclitaxel, could be the development of P-glycoprotein drug-efflux pump that practically abolish the effect of Paclitaxel, in contrary, this pump doesn’t work for epothilone, that gave to epothilones an extraordinary privilege to be a powerful drug for the multidrug resistant tumors [42]. Ixabepilone has been reported as potent cytotoxic agent across a panel of multidrug-resistant cell lines: breast, colon, and lung with the majority of IC_50_ values between 1.4 and 45 nm [7]. The derivative of epothilone B, ABJ-879, has exhibited greater potency than paclitaxel across a panel of human tumor cell lines [5, 9, 55].

The effect of extracted epothilone B on the tubulin polymerization was assessed using the fluorescence-based tubulin polymerization assay, compared to Paclitaxel as positive control. The purified epothilone displayed a significant anti-tubulin polymerization activity by about 4 folds higher than Paclitaxel, as revealed from the IC_50_ values. Microtubule-targeting “antimitotic drugs”, disrupts the microtubule of tumor cells, inducing the cell cycle arrest in G2/M phase and the subsequent formation of abnormal mitotic spindles [56]. Therefore, tubulin polymerization inhibitors have become an important class of antineoplastic agents. There are two groups of tubulin/microtubule-targeting drugs: microtubule stabilizing agents “enhance the microtubule polymerization” (Epothilones and Paclitaxel) and microtubule destabilizing agents “inhibits microtubule polymerization” (Colchicine and Vinca alkaloids) [6, 57, 58]. Three major binding sites on α, β-tubulin subunits have been identified as taxane [59, 60], vinca alkaloid [61], and colchicine-binding sites [62]. Paclitaxel and Epothilone are known as microtubule inhibitors of the taxane-site, and Vinblastine are vinblastine-site inhibitors [3, 4]. Interestingly, in contrary to paclitaxel, Epothilone retained full activity against cancer cells overexpressing the drug efflux pump P-glycoprotein (Pgp) or harboring tubulin mutations [55].

The purified *A. fumigatus* epothilone has a strong effect on the migration ability of the HepG2 tumor cells as reveled from the wound healing assay, compared to the negative control cells (untreated cells), and positive control cells (Doxorubicin treated). The wound closure of the HepG2 monolayer cells was noticeably suppressed with the purified epothilone, ensuring the interference with the cellular regeneration, cell divisions, and matrix formation of the tumor cells. Similar result authenticates the profound role of epothilone in inhibition of wound healing by inhibition of migration of meningeal fibroblasts by drastically changing of their microtubular network [63]. Consistently, epothilone has a significant effect on inhibition of wound healing of MCF7 cells, after exposure to the IC_50_ value, the wound closure was only 40% of the control width.

The effect of the extracted epothilone from *A. fumigatus* on the apoptosis process of the HepG2 cells was assessed. A significant shift of the normal cells to the apoptotic stages in response to epothilone treatment compared to control cells*.* The total ration of apoptosis of HepG2 cells was increased by ~ 20 times responsive to epothilone treatment compared to control. Coincidently, the antiproliferative activity of Epo A and Epo B for the SKOV-3 cell line was higher than that of Paclitaxel by 5-6 folds, with an induced time course-dependent apoptosis [64]. Apoptosis is the main type of programmed cell death, involves a series of biochemical events leads to cellular morphological changes and ultimate cell death, so apoptotic stimuli is an profound factor in cancer biology [65]. Taxanes and epothilones have been widely reported to promote apoptosis in a variety of cell types [65]. The current apoptotic analysis of HepG2 in response to *A. fumigatus* epothilone was evaluated with annexin V-FITC-propidium iodide stain that mainly reacts with phosphatidylserine of apoptotic cells. Phosphatidylserine (PS) externalization is a consequence of the impairment of plasma membrane lipid asymmetry, that regarded as an early apoptosis [66]. Similar results were reported for the Epo B, that inducing apoptosis in a dose dependent-manner of the non-small cell lung cancer cells [67]. The effect of *A. fumigatus* epothilone as a potent apoptotic stimulus of HepG2, could be associated with some apoptotic features namely chromatin condensation, nuclear fragmentation, fragmented DNA, and apoptotic bodies formation [6, 7]. The cell cycle analysis of the HepG2 in response to the *A. fumigatus* epothilone was performed by propidium iodide at the IC_25_ values of extracted epothilone. Necrotic cells which have lost membrane integrity showed red staining (PI) throughout the nucleus. Noticeably, the highest growth arrest of the HepG2 cells was reported at the G0-G1 phase in response to treatment with *A. fumigatus* epothilone compared to the without treatment cells. The growth arrest of the HepG2 tumor cells at the S and G2/M phases in response to epothilone treatment was relatively similar to the negative control cells. Overall, the epothilone extracted from *A. fumigatus* had a significant activity at the G0-G1 cell cycle as revealed from the maximum growth arrest, compared to the other phases of the cell cycle.

In conclusion, the epothilone productivity by *A. fumigatus* has been assessed using various solid substrates under solid state fermentation conditions. Among the different tested substrates, orange peels as solid substrate, gave the maximum productivity of epothilone by *A. fumigatus*. The productivity of epothilone by *A. fumigatus* has been optimized by Plackett-Burman design with an obvious increase to the yield of epothilone by about 2 folds than the corresponding medium under submerged conditions. The chemical structure of the purified epothilone has been verified from the chromatographic analysis and spectroscopic analysis by LC-MS/MS, gave the same molecular fragmentation pattern to the standard epothilone. The antiproliferative activity of the extracted epothilone gave a highest activity against the tested HepG2, MCF7, WI38 and Pancl cell lines compared to the Paclitaxel as standard drug. The activity of purified epothilone against tubulin polymerization was about 4 folds higher than paclitaxel, with significant activity against the wound healing of HepG2 cells. As well as, the purified epothilone of *A. fumigatus* had a dramatic activity in inducing the early apoptosis of HepG2 cells and arresting the cells at the G0-G1 phase. This is the first report, emphasizing the possibility of *A. fumigatus* grown on orange peels as solid substrate for production of epothilone that could be a novel platform for commercial production of epothilone B.

## Declarations

### Data availability

All datasets generated for this study are included in the article.
